# Priority setting and health policy and systems research

**DOI:** 10.1186/1478-4505-7-27

**Published:** 2009-12-04

**Authors:** Michael K Ranson, Sara C Bennett

**Affiliations:** 1 Alliance for Health Policy and Systems Research, World Health Organization, 20 Avenue Appia - 1211 Geneva 27, Switzerland

## Abstract

Health policy and systems research (HPSR) has been identified as critical to scaling-up interventions to achieve the millennium development goals, but research priority setting exercises often do not address HPSR well. This paper aims to (i) assess current priority setting methods and the extent to which they adequately include HPSR and (ii) draw lessons regarding how HPSR priority setting can be enhanced to promote relevant HPSR, and to strengthen developing country leadership of research agendas.

Priority setting processes can be distinguished by the level at which they occur, their degree of comprehensiveness in terms of the topic addressed, the balance between technical versus interpretive approaches and the stakeholders involved. When HPSR is considered through technical, disease-driven priority setting processes it is systematically under-valued. More successful approaches for considering HPSR are typically nationally-driven, interpretive and engage a range of stakeholders. There is still a need however for better defined approaches to enable research funders to determine the relative weight to assign to disease specific research versus HPSR and other forms of cross-cutting health research.

While country-level research priority setting is key, there is likely to be a continued need for the identification of global research priorities for HPSR. The paper argues that such global priorities can and should be driven by country level priorities.

## Introduction

Failing or inadequate health systems are one of the main barriers to scaling-up interventions towards the achievement of the millennium development goals [1] and there is increasing consensus about the need for global action on health systems [2]. However international research funding has been devoted largely to upstream health research -- basic science, biomedical research related to specific diseases and technical intervention development [3]. A stronger body of knowledge about which health policy and health system strengthening strategies are effective, and which are not, is urgently needed [4,5].

Research priority setting is acknowledged to be a key function of national health research systems and is perceived to be an important process in terms of ensuring the alignment of research funding with national evidence needs [6]. Ideally health policy and systems research (HPSR) priorities would emerge through priority setting processes. However priority setting for health research is often not performed well - or not performed at all. A survey by the WHO of more than 550 policy makers and almost 1, 900 researchers in 13 low- and middle-income countries found that about a third of policy-makers, researchers and users of research interviewed said that there was either no rational process to set health research priorities in their country or that they were unaware of how priorities were identified or set [7].

Much of HPSR is context specific and thus the role of national health authorities in setting HPSR priorities is particularly important. However, in many developing countries, significant funding for HPSR comes from external sources, and while HPSR research priorities should be set at the national level, there is likely to be a continued need for information about HPSR priorities at the global level. Indeed there are several current developments for which sound priority setting processes are required. For example, the Intergovernmental Working Group on Public Health, Innovation and Intellectual Property prepared a global strategy and plan of action on essential health research to address conditions affecting developing countries disproportionately. The first element of the strategy involves prioritizing research and development needs, in part by "formulating explicit prioritized strategies for research and development at country and regional and inter-regional levels" [8]. In light of this, as well as the other factors described above there is an urgent need for the development of research priority setting processes for HPSR that reflect national research needs but also inform global agendas.

A substantial amount of previous work on priority setting processes for health research has been conducted [6,9-11], but this article argues that there is still a lack of clarity as to how to ensure that HPSR priorities are adequately reflected on both national and global research agendas. The purpose of this paper is:-

• To explore current priority setting methods and the extent to which they adequately include HPSR

• To draw lessons regarding how HPSR priority setting can be enhanced to strengthen developing country leadership of research agendas, and to promote relevant HPSR

This paper does not set out to provide a comprehensive review of previous health research priority setting processes, which can be found elsewhere [9-12]. It should be noted that while the focus of this paper is HPSR, many of the arguments apply equally to other types of research--for example research on the social determinants of health--that are not disease-specific.

## Overview of priority setting approaches and HPSR

Priority setting processes can be distinguished by a number of inter-linked factors including:-

**• *Level: ***they may aim to set research priorities at global, national, sub-national or institutional levels.

**• *Comprehensiveness***: some priority setting processes focus on relatively narrow sub-sets of research questions (what are the key research questions in the field of malaria control, or health workforce for example), whereas others aim to set research priorities for the whole of the health sector, others still may situate health research priorities within the broader context of scientific investment and research priorities in other fields.

**• *The balance between technical versus interpretive approaches***: priority setting processes within the health sector may be predominantly technical in nature, dominated by quantifiable epidemiologic or other needs and costs data, or more interpretive assessments, dominated by the consensus views of informed participants [13].

**• *Stakeholders***- Those included in consultative process may be primarily researchers, or include a broader audience of research funders, research users and communities.

So how in practice is HPSR typically included in research priority setting processes? Table 1 categorises previous priority setting exercises that have encompassed HPSR, based on whether they aimed to establish priorities at the global, national or institutional level. In broad terms, HPSR issues may be incorporated into a priority setting exercise either through the lens of a specific disease(s), or as a category separate from specific diseases.

**Table 1:** Select priority setting processes categorized by level and disease specificity

	Disease specific	HPSR considered alone or separately
**Global level**	• Child Health and Nutrition Research Initiative -- Childhood pneumonia and diarrhoea • Global Forum for Health Research -- use of Combined Approach Matrix for research on schizophrenia, indoor air pollution	• The Ad Hoc Committee on Health Research Relating to Future Intervention Options [26] • Task Force on Health Systems Research [17]

**National level**	• Child Health and Nutrition Research Initiative -- South Africa • Global Forum for Health Research -- use of Combined Approach Matrix for: diarrhoeal diseases research in India; research for the prevention and control of noncommunicable diseases in Pakistan	• Brazil and Malaysia national priority setting • England's National Service Delivery & Organisation research and development program [13,27] • Canada, consortia including Canadian Health Services Research Foundation [28]

**Institutional level**	• Special Programme for Research & Training in Tropical Diseases (application of Combined Approach Matrix)	• World Health Organization Advisory Committee on Health Research [29]

Where HPSR issues have been looked at through the lens of a specific disease, the priority setting exercise has typically been more technical versus interpretive in nature, and has been at least partially driven by disease burden. Such priority setting processes generally determine what burden of disease may be averted by:-

1. Developing new drugs and technologies

2. Improving existing drugs and technologies

3. Extending the uptake of existing interventions.

Health systems research falls into the third of these categories, and may, through this route be prioritized.

An example of a predominantly disease-driven approach to priority setting is the Combined Approach Matrix (CAM) [14]. The Matrix helps to organize and summarize information about a particular disease and the interventions available to combat it. The matrix categorizes this information according to five "economic dimensions" (e.g. disease burden and cost-effectiveness of interventions) and four "institutional dimensions" representing the various levels at which interventions can be implemented. The methodology has been applied at institutional, national and global levels.

Like other disease-specific approaches, the CAM methodology does not serve health policy and systems research well. Many health systems research questions stand to provide benefits for multiple different diseases. Linking health systems research questions to specific diseases, rather than seeing them in total, results in these topics being systematically de-prioritized and contributes to the fragmentation of health systems research. For example, the research questions and methods to address the norms and training that underlie prescription practice will be very similar whether one is talking about diarrhoea or malaria, HIV/AIDS or tuberculosis. Combining research that looks at this question for a variety of different diseases will be both conceptually more sound and more cost-effective. Disease-specific approaches occasionally lead to research priorities that are difficult to interpret. For example, application of the Child Health and Nutrition Research Initiative (CHNRI) method in South Africa resulted in a very high ranking for "health policy and systems research to improve hand washing with soap" [15]: the nature of the exact research question is difficult to understand.

An alternative approach to the identification of health policy and systems research priorities has been to focus on HPSR questions separate from other disease-specific questions, and typically to use a more interpretive approach. This strategy was adopted for example by the Task Force on Health Systems Research (established in 2003), which aimed to develop a global research agenda to support the attainment of the Millennium Development Goals [16,17]. Previous approaches to priority setting in health research were reviewed for their applicability to health systems research. With inputs from WHO staff and other experts, a tentative research agenda was developed comprising 12 topic areas that were intended to cover the important barriers to improving health systems performance. The Task Force then undertook a consultative process involving several WHO regional meetings, an article in The Lancet, a presentation at the Ministerial Summit in Health Research in Mexico, and the extensive circulation of the preliminary research agenda using e-mail discussion lists to collect and collate feedback on the tentative agenda.

HPSR issues were also dealt with separately in the national health research priority setting process in Malaysia [18]. Experts first identified eleven broad topic areas. Eight of these areas related to the leading causes of burden of disease (e.g. cancer, mental illness) and three topic areas dealt with cross-cutting issues, including "Health Policy and Systems". Within the disease-specific topic areas, information gaps were identified using the CAM methodology. However, the CAM methodology could not be applied to the cross-cutting topic areas -- in order to identify priorities within each of the these three topic areas selected groups of stakeholders (mostly experts) prioritized the potential research topics using agreed criteria for rank-ordering. These priority lists were reviewed and validated by a broader group of approximately 600 stakeholders during a national conference held in July, 2006.

Priority setting processes that deal with HPSR issues separate from disease-specific questions also have their weaknesses. First, they create some difficulties for funders in that they give no indication as to the importance of HPSR topics relative to disease specific topics and other forms of cross-cutting research. Second, the results are likely to be determined by the range and nature of stakeholder groups consulted in the process. These challenges are addressed in the final section, below.

## Approaches to policy-maker driven global priority setting processes

While, the Working Group on Priority Setting (2000) argued that priority setting processes should be demand driven, and involve multiple different types of informational inputs as well as multiple stakeholder perspectives, as described above most approaches to global priority setting have taken place at the global level and have not been driven by country level priorities. The extent to which the product of such global level priority setting processes really reflects country level needs is unclear. One exception that has tried to drive global priorities based upon the evidence needs articulated by policy makers in low and middle income countries is recent work by the Alliance for HPSR. This programme of work aims to identify high priority, tractable health systems research questions in three thematic areas: human resources for health [19], health system financing [20] and the role of the non-state sector in service provision [21]. These three broad areas were identified by the Task Force on Health Systems Research as being important, and this view was reinforced by both national and global level stakeholders in the Alliance HPSR who argued that the Alliance should focus its work on these themes. The overall purpose of the process was to inform and influence the funding strategies of global level research funders, and to promote funding alignment with policy maker needs, as well as to stimulate more coordinated researcher interest in high priority questions.

The Alliance used three steps in the priority setting process. The first, and principal input into the priority-setting process were reports from four regional reports that identified and ranked common policy concerns and research priorities in each of the three thematic areas. These studies, implemented by regional institutions and networks drew on: (i) key informant interviews conducted with policy makers, researchers, community and civil society representatives in each of twenty-four countries; and (ii) review of documents pertaining to previous priority setting processes, where available.

As a second step, at the global level, overviews of existing literature reviews and systematic reviews were conducted in order to ascertain the supply of research, and analysis of regional and country reports was undertaken to identify research priorities that cut across multiple countries. For each of the thematic areas, the commonly identified priority research questions were compared with what was available in the existing literature to come up with a short list of (approximately 20) research questions.

Third, theme-specific workshops at the international level were conducted, where key stakeholders (primarily researchers, but also policy makers, research funders, and representatives of international organizations) were asked to clarify and discuss the emerging priority research questions and rank them. Table 2 lists the research questions that ranked highest at these workshops.

**Table 2:** Top-ranked research questions: human resources for health, health systems financing and the non-state sector

	Human resources for health	Health system financing	Non-state sector
**1st**	To what extent do financial and non-financial incentives work in attracting and retaining qualified health workers to under-serviced areas?	How do we develop and implement universal financial protection?	How can the government create a better environment to foster non-state providers in the achievement of health systems outcomes?

**2nd**	What is the impact of dual practice (i.e. practice by a single health care worker in both the public and the private sectors) and multiple employment? Are regulations on dual practice required, and if so how should they be designed and implemented?	What are the pros and cons of the different ways of identifying the poor?	What is the quality and/or coverage of health care services provided by the non-state sector for the poor?

**3rd**	How can financial and non-financial incentives be used to optimize efficiency and quality of health care?	To what extent do health benefits reach the poor?	What types of regulation can improve health systems outcomes, and under what conditions?

**4th**	What is the optimal mix of financial, regulatory and non-financial policies to improve distribution and retention of health workers?	What are the pros and cons of implementing demand-side subsidies?	How best to capture data and trends about private sector providers on a routine basis?

**5th**	What are the extent and effects of the out-migration of health workers and what can be done to mitigate problems of out-migration?	What is the equity impact of social health insurance and how can it be improved?	What are the costs and affordability of the non-state sector goods and services relative to the state sector? And to whom?

This exercise was innovative in terms of building global priorities upon country priority setting processes and ensuring that country policy maker needs are reflected in the research priorities identified at the global level. An iterative process was used to generate the list of questions, favouring those that were expressed by respondents in more than one country, and increasing the generalizability to other developing countries. The process used in all three steps of the study have been carefully documented and described, and region- (and to a lesser extent country-) level collaborators have been involved in global-level priority ranking and report writing.

The primary weakness of this approach is that the phased nature of the process means that it can be resource intensive and time consuming - the latter is particularly a problem given the fact that research priorities may be time sensitive. Such problems could be addressed through clearer and better coordinated structures for priority setting that help make priority setting a routine function of health research systems and ensure that global processes build upon country processes in a timely fashion. Such work also requires qualitative research skills in order to avoid biases in the selection of stakeholders, and the relative weight allotted to these stakeholders in data collection and analyses.

## Developing better processes for prioritizing HPSR issues

Setting priorities for HPSR through a disease specific lens does not seem a rational approach, and it is likely to systematically undervalue the contribution of HPSR. Priority setting processes for HPSR are best when qualitative in nature.

While prior global priority setting processes for HPSR have relied upon relatively limited consultative processes that have taken place primarily at the international level, there appears to be a strong rationale for using country priority setting processes to inform the development of global priorities. There is likely to be a continued demand for the identification of global research priorities due both to the nature of funding for HPSR and the continued reliance on international funding sources, but also an ongoing need for multi-country research studies that yield more generalizable conclusions. The development and routine implementation of global processes that build global research priorities upon country research priorities, can help to stimulate more routine country-level priority setting processes, and also ensure that funded research does indeed match the needs of policy makers.

A further advantage of nationally-driven interpretive approaches to HPSR priority setting is that they provide opportunities for engaging stakeholders in this field of research. Lomas *et al*. comment that "these exercises are an important step in the ongoing 'linkage and exchange' between those who fund and conduct applied health services research and the stakeholders whom the research aims to influence" [p. 383, [13]]. Other papers that have explored methods for closing the "know-do gap" (or evidence-based policy and practice) have similarly highlighted the potential importance of participatory processes for establishing research and information needs [22,23].

While there are significant advantages to setting HPSR priorities through the type of nationally-driven, interpretive approach discussed here, our analysis suggests a need for further thought on a number of issues. Specifically:-

- *Development of mechanisms to determine the relative importance of research priorities established through different types of priority setting exercises*. The use of separate processes to identify HPSR priorities and disease-specific priorities creates difficulties for research funders in terms of determining the relative weight to assign to disease-specific research versus HPSR (and other forms of cross-cutting research). While there are potential methods to address this question, they have not been applied in the field of global health research. For example, the Copenhagen Consensus exercise aimed to set priorities across a broad range of activities (including research) for improving the lives of people living in developing countries [24,25]. Proposals were compared across sectors and types of intervention using cost-benefit analysis, that assessed the ratio of social benefit to cost. A similar quantitative approach could be applied to different broad areas of health research. Alternatively, and perhaps more straightforwardly, priorities across different types of research could be established through qualitative, consultative approaches, as demonstrated for example by the Australian National Research Priorities exercise that through a largely consultative process identified four research themes across different sectors, that were perceived to be of long term importance to Australia.

- *Systematic investigation and development of guidance on howstakeholder engagement affects research priority identification*. The final results of an interpretive research priority setting exercise, are likely to depend significantly on the range of stakeholders consulted during the process. The COHRED Working Group on Priority Setting noted that "The participation of a broadened spectrum of stakeholders helps to identify research needs, technical and financial capabilities, and the values and ethics of a given society" [[6], pg 132]. Conversely, not bringing certain groups - such as policy makers and civil society organisations into the priority setting process may contribute to the neglect of certain health research fields, including HPSR. While this is understood in general terms, the relationship between stakeholder involvement and the type of priorities identified has not been well documented. The relative weight or priority placed upon the viewpoints of different stakeholders can be adjusted according to the objective of the exercise and whose voice needs to be heard [13], but there is no clear set of principles guiding how best to do this. Finally, the outcome of an interpretive research priority setting exercise will depend also on whether data on health system performance (and constraints) is used as part of the process, and if so, how this is incorporated and weighted relative to the judgement of participants.

- *Information needs of target audiences *- Priority setting exercises need to take their target audience into account [12]. The appropriate level and comprehensiveness of research priority setting exercises depend upon who it is ultimately anticipated will fund the research. Different funders approach research investment in different fashions. For example some determine relatively narrow topics on which they wish to invite proposals, whereas others give greater scope so as to support investigator-driven enquiry. There is no mapping of, or systematic enquiry into how research funders would like to see research priorities presented.

- *Understanding the implications of HPSR capacity constraints for the identification of research priorities*. The fact that few priority setting processes properly address health systems research is one reason why funding for the field has been relatively limited. However another factor undermining funding for HPSR, that could perhaps be better taken account of in priority setting processes, is the weak capacity to conceptualize, develop, and implement HPSR in low and middle income countries. Good analyses are needed of how limited research capacity shapes the nature of research priorities, and how HPSR priority setting could also address capacity strengthening so as to strike a balance between investments in direct research funding and in capacity development.

## Competing interests

The authors declare that they have no competing interests.

## Authors' contributions

MKR and SCB conceived of the study and designed the study. MKR conducted the literature review. MKR and SCB wrote the paper. Both authors have read and approved the final manuscript.

## References

[B1] World Health Organization Everybody's Business: Strengthening Health Systems to Improve Health Outcomes, WHO's Framework for Action 2007 Geneva: WHO

[B2] Reich M Takemi K Roberts M Hsiao W Global action on health systems: a proposal for the Toyako G8 summit The Lancet 2008 371 9615 865 9 10.1016/S0140-6736(08)60384-0 18328932

[B3] Leroy JL Habicht JP Pelto G Bertozzi SM Current priorities in health research funding and lack of impact on the number of child deaths per year American journal of public health 2007 97 2 219 23 10.2105/AJPH.2005.083287 17194855 PMC1781402

[B4] Travis P Bennett S Haines A Pang T Bhutta Z Hyder AA Pielemeier NR Mills A Evans T Overcoming health-systems constraints to achieve the Millennium Development Goals Lancet 2004 364 9437 900 6 10.1016/S0140-6736(04)16987-0 15351199

[B5] Murray CJ Frenk J Evans T The Global Campaign for the Health MDGs: challenges, opportunities, and the imperative of shared learning Lancet 2007 370 9592 1018 20 10.1016/S0140-6736(07)61458-5 17889229

[B6] Working Group on Priority Setting Priority setting for health research: lessons from developing countries Health policy and planning 2000 15 2 130 6 10.1093/heapol/15.2.130 10837035

[B7] World Health Organization World Report on Knowledge for Better Health: Strengthening Health Systems 2004 Geneva: WHO

[B8] World Health Organization61st World Health Assembly, Agenda item 11.6. Global strategy and plan of action on public health, innovation and intellectual property 2008 WHO http://apps.who.int/gb/ebwha/pdf_files/A61/A61_R21-en.pdf (accessed on 12 November 2009)

[B9] Global Forum for Health Research 10/90 Report on Health Research 2003-2004 2004 Geneva: GFHR

[B10] Rudan I Gibson J Kapiriri L Lansang MA Hyder AA Lawn J Darmstadt GL Cousens S Bhutta ZA Brown KH Hess SY Black M Gardner JM Webster J Carneiro I Chandramohan D Kosek M Lanata CF Tomlinson M Chopra M Ameratunga S Campbell H El Arifeen S Black RE Setting priorities in global child health research investments: assessment of principles and practice Croatian medical journal 2007 48 5 595 604 17948946 PMC2205967

[B11] Nuyens Y Setting priorities for health research: lessons from low- and middle-income countries Bulletin of the World Health Organization 2007 85 4 319 21 10.2471/BLT.06.032375 17546314 PMC2636318

[B12] Alliance for Health Policy and Systems Research Strengthening health systems: the role and promise of policy and systems research 2004 Geneva: AHPSR

[B13] Lomas J Fulop N Gagnon D Allen P On being a good listener: setting priorities for applied health services research The Milbank quarterly 2003 81 3 363 88 10.1111/1468-0009.t01-1-00060 12941000 PMC2690239

[B14] Ghaffar A de Francisco A Matlin S The combined approach matrix: a priority setting tool for health research 2004 Geneva: Global Forum for Health Research

[B15] Tomlinson M Chopra M Sanders D Bradshaw D Hendricks M Greenfield D Black RE El Arifeen S Rudan I Setting priorities in child health research investments for South Africa PLoS medicine 2007 4 8 e259 10.1371/journal.pmed.0040259 17760497 PMC1952202

[B16] Task Force on Health Systems Research The Millennium Development Goals will not be attained without new research addressing health system constraints to delivering effective interventions 2005 Geneva: World Health Organization

[B17] Task Force on Health Systems Research Informed choices for attaining the Millennium Development Goals: towards an international cooperative agenda for health-systems research Lancet 2004 364 9438 997 1003 10.1016/S0140-6736(04)17026-8 15364193

[B18] Hamid MA Health Research Priorities: Malaysia. Priority Setting Methodologies in Health Research (10-11 April 2008) 2008 WHO, Geneva in press

[B19] Ranson MK Chopra M Munro S Dal Poz M Bennett S What are research priorities for human resources for health in low and middle income countries? Bulletin of the World Health Organization 2010 in press 10.2471/BLT.09.066290 PMC2878144 20539857

[B20] Ranson MK Law TJ Bennett S Establishing health system financing research priorities in developing countries using a participatory methodology Soc Sci Med 2010 in press 10.1016/j.socscimed.2010.01.051 20378228

[B21] Walker D Champion C Hossain S Wahed T Gazi R Koehlmoos TP Asiimwe D Ranson KM Bennett S Alliance HPSR Technical Paper: Establishing non-state sector research priorities in developing countries using a participatory methodology 2009 http://www.who.int/alliance-hpsr/researchsynthesis/Alliance_HPSR-nonstatesectorprioritysettingreport.pdf

[B22] van Kammen J de Savigny D Sewankambo N Using knowledge brokering to promote evidence-based policy-making: The need for support structures Bulletin of the World Health Organization 2006 84 8 608 12 10.2471/BLT.05.028308 16917647 PMC2627440

[B23] Lavis JN Lomas J Hamid M Sewankambo NK Assessing country-level efforts to link research to action Bulletin of the World Health Organization 2006 84 8 620 8 10.2471/BLT.06.030312 16917649 PMC2627430

[B24] Copenhagen Consensus Putting the world to rights The Economist 2004

[B25] Copenhagen Consensus Center Copenhagen Consensus 2008 http://www.copenhagenconsensus.com/Default.aspx?ID=788 [cited 2008 21 May]

[B26] Ad Hoc Committe on Health Research Relating to Future Intervention Options. Investing in health research and development 1996 Geneva: World Health Organization

[B27] Fulop N Allen P National Listening Exercise: Report of Findings NHS Service Delivery & Organisation 2000 http://www.sdo.lshtm.ac.uk/files/adhoc/listening-exercise-2000.pdf (accessed 15 April 2008).

[B28] Canadian Health Services Research Foundation Listening for Direction III: Preliminary results 2008 http://www.chsrf.ca/other_documents/listening/direction-3-results_e.php (accessed 15 April 2008)

[B29] Advisory Committee on Health Research Health Research Strategy for Health for All by the Year 2000: Report of a Subcommittee of the ACHR 1986 Geneva: WHO

